# Supplementary material for: Facile Synthesis of Functionalised Hyperbranched Polymers for Application as Novel, Low Viscosity Lubricant Formulation Components
Source: Polymers (Basel). 2022 Sep 14;14(18):3841. doi: 10.3390/polym14183841 (PMC9501064; doi:10.3390/polym14183841)
Supplement: Supplementary file 1 [file polymers-14-03841-s001.zip › polymers-1888288-supplementary.pdf]

Facile Synthesis of Functionalised Hyperbranched Polymers for Application as Novel, Low Viscosity Lubricant Formulation Components

Sophie R. Goodwin,[1] Amy Stimpson,[1] Richard Moon, [1] Lauren Cowie,[2] Najib Aragrag,[2,#] Sorin V. Filip,[2] Andrew G. Smith,[2] and Derek J. Irvine[1]

[1] Centre for Additive Manufacturing, Faculty of Engineering, University of Nottingham, University Park, Nottingham, NG7 2RD, UK.
[2] bp Applied Sciences, Innovation & Engineering, Technology Centre, Pangbourne, UK
[#] Current Address: ExxonMobil, Hermeslaan 2, Machelen, Flemish Region 1831, BE

Keywords: Hyperbranched polymers, Catalytic chain transfer, Lubricants, Low Viscosity,

Corresponding authors contact details: Tel.: +44(0)1159514088 E-mail address: derek.irvine@nottingham.ac.uk

a)

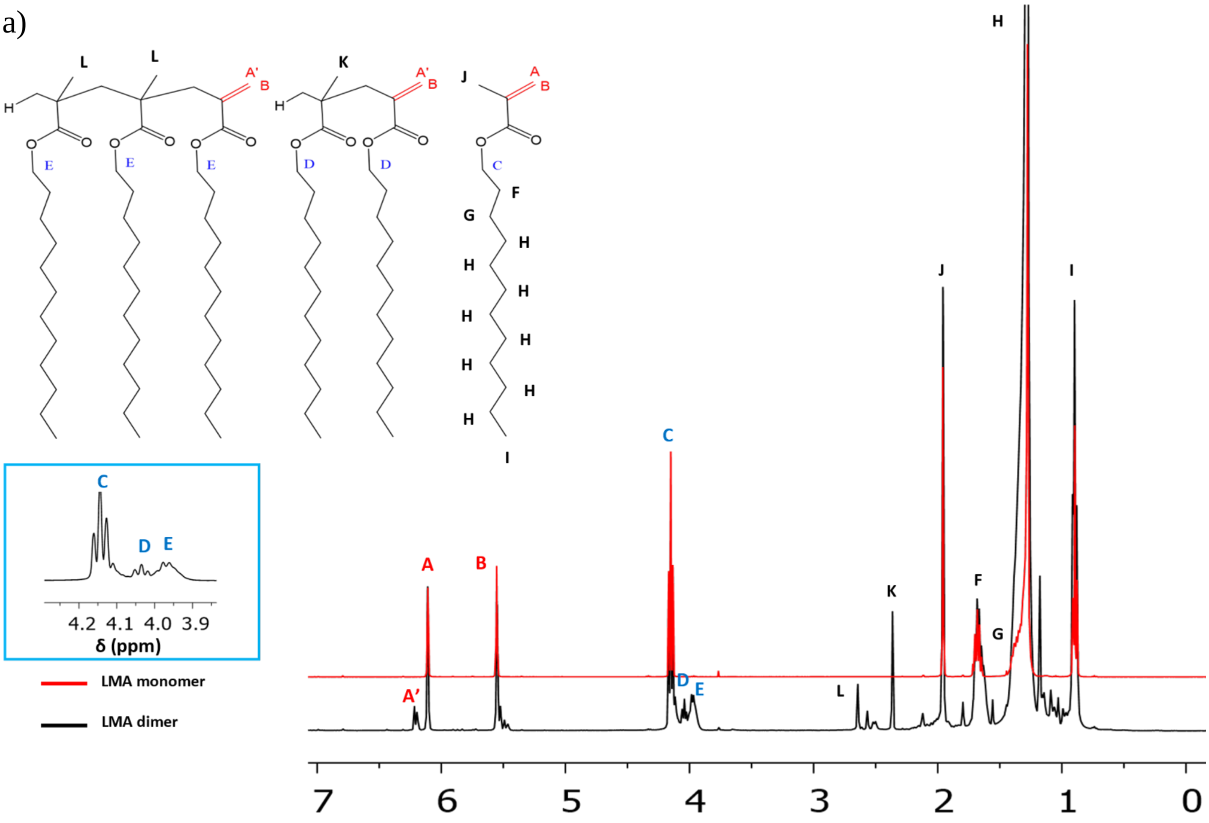

b)

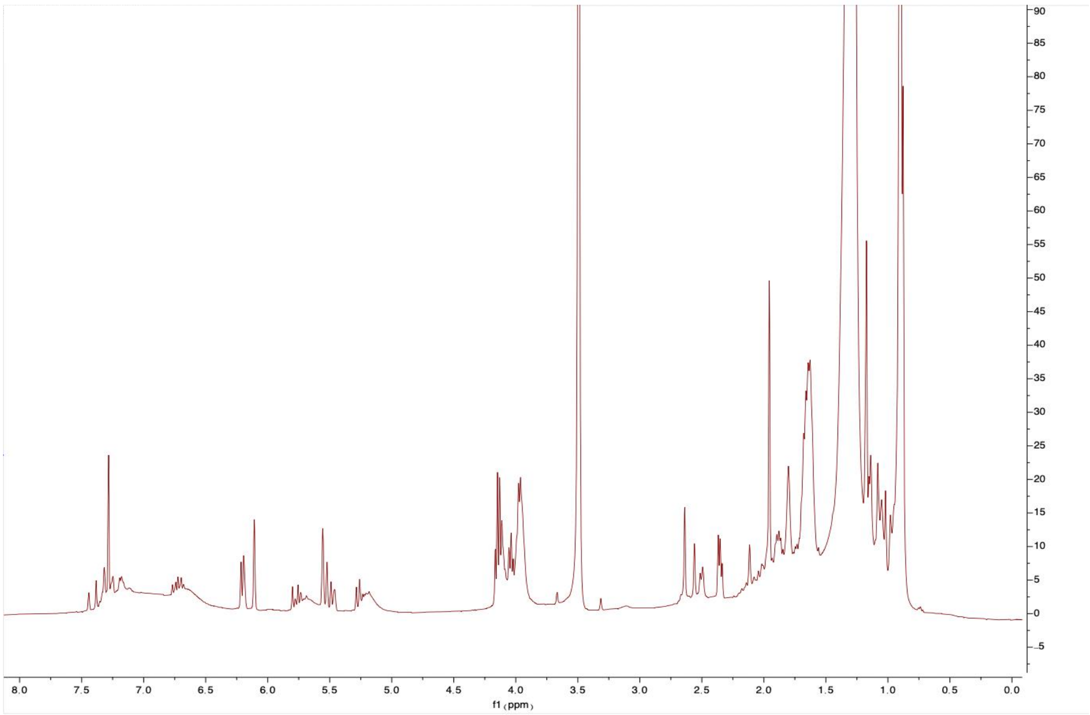

Figure S1. H^1 NMR spectra of a) LMA monomer and oligomer mixture & b) a typical block-like DVB-co-LMA hyperbranched polymer

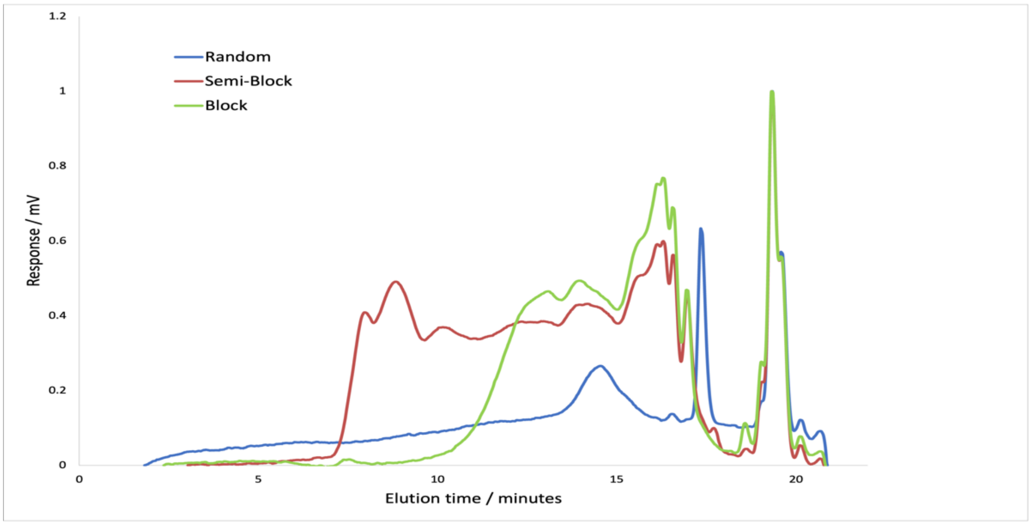

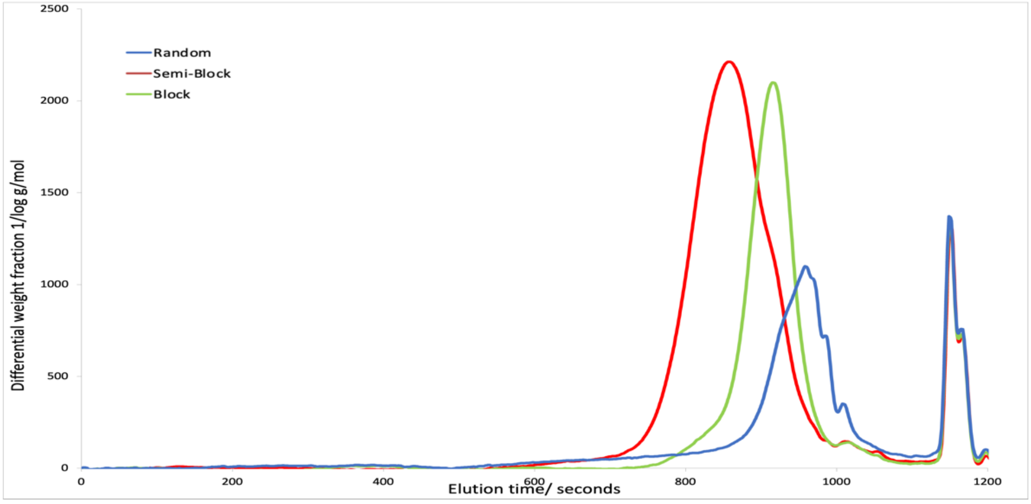

Figure S2. GPC data for the materials from the optimised hyperbranching synthesis methodologies for both solvent/base oil solubility and lubricity application testing. The upper chromatogram contains the IR detector data the lower the light scattering data for the same samples.

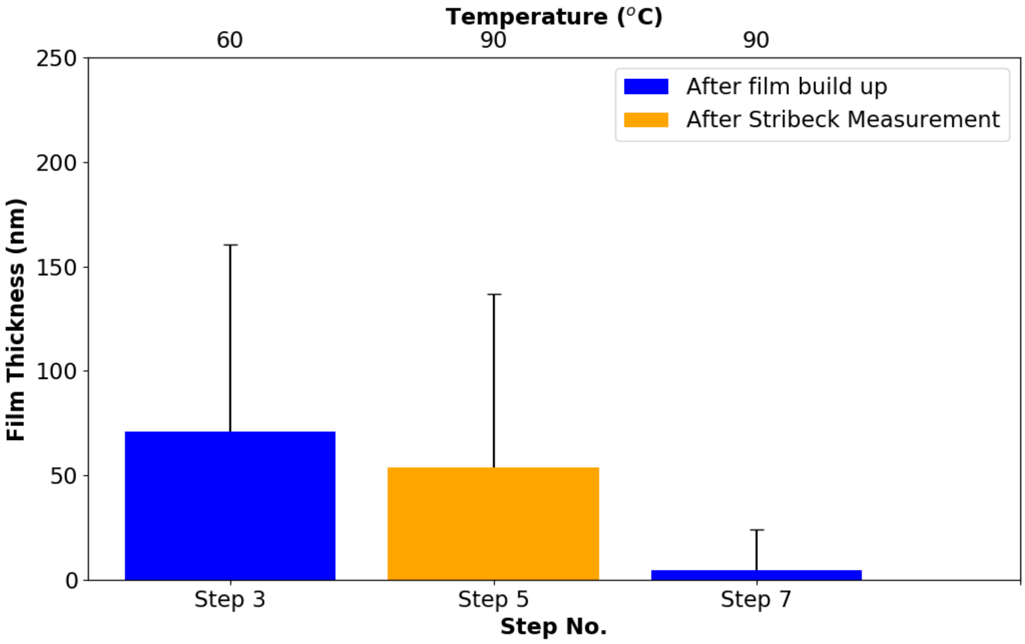

Figure S3. A visual representation of the data in Table 3, Entries B-D. It contains the plot of the film thickness in nm for these entries. The overall data in Figure S1 and Table 3 represent the summary of the film thickness data obtained from interferometry for a pure oil solution. The blue shading indicates that the measurement was made after film build up (i.e. following a period with no change of temperature or rolling speed). The orange shading indicates that the measurement was made after a Stribeck Curve measurement was made.

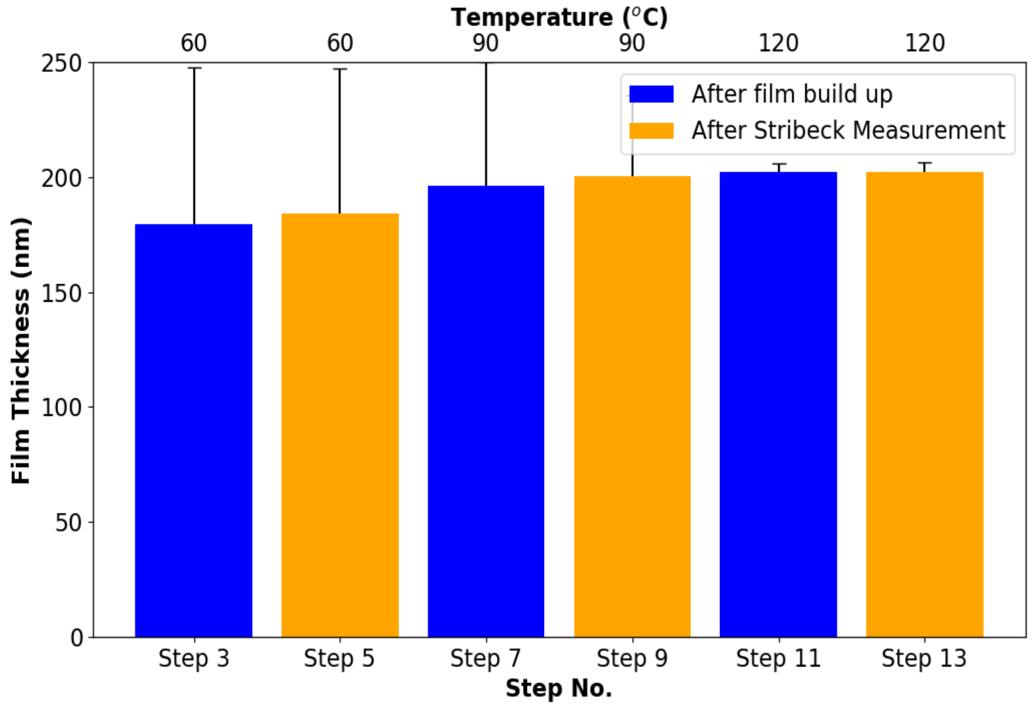

Figure S4. A visual representation of the data in Table 4, Entries B-G. It contains the plot of the film thickness in nm for these entries. The overall data in Figure S2 and Table 3 represent the summary of the film thickness data obtained from interferometry for a 1 wt % semi-block-like HB polymer/base oil formulation. The blue shading indicates that the measurement was made after film build up (i.e. following a period of constant temperature and rolling speed). The orange shading indicates that the measurement was made after a Stribeck Curve measurement was made.

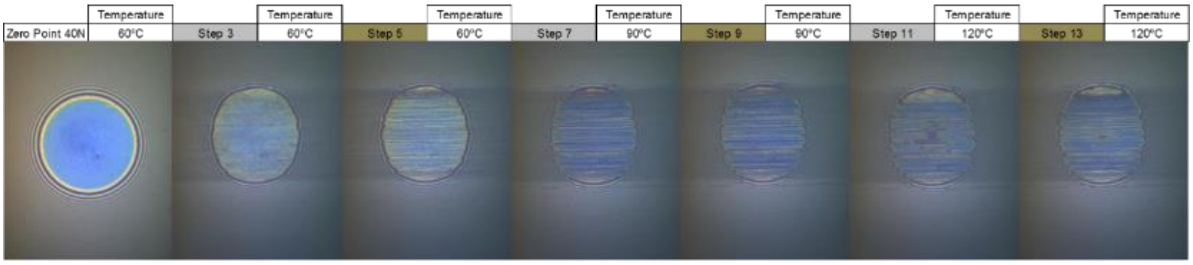

(A) (B) (C) (D) (E) (F) (G)

Figure S5. Interferometry images for an experiment utilising a 1 wt% Block-like HB polymer in oil solution.

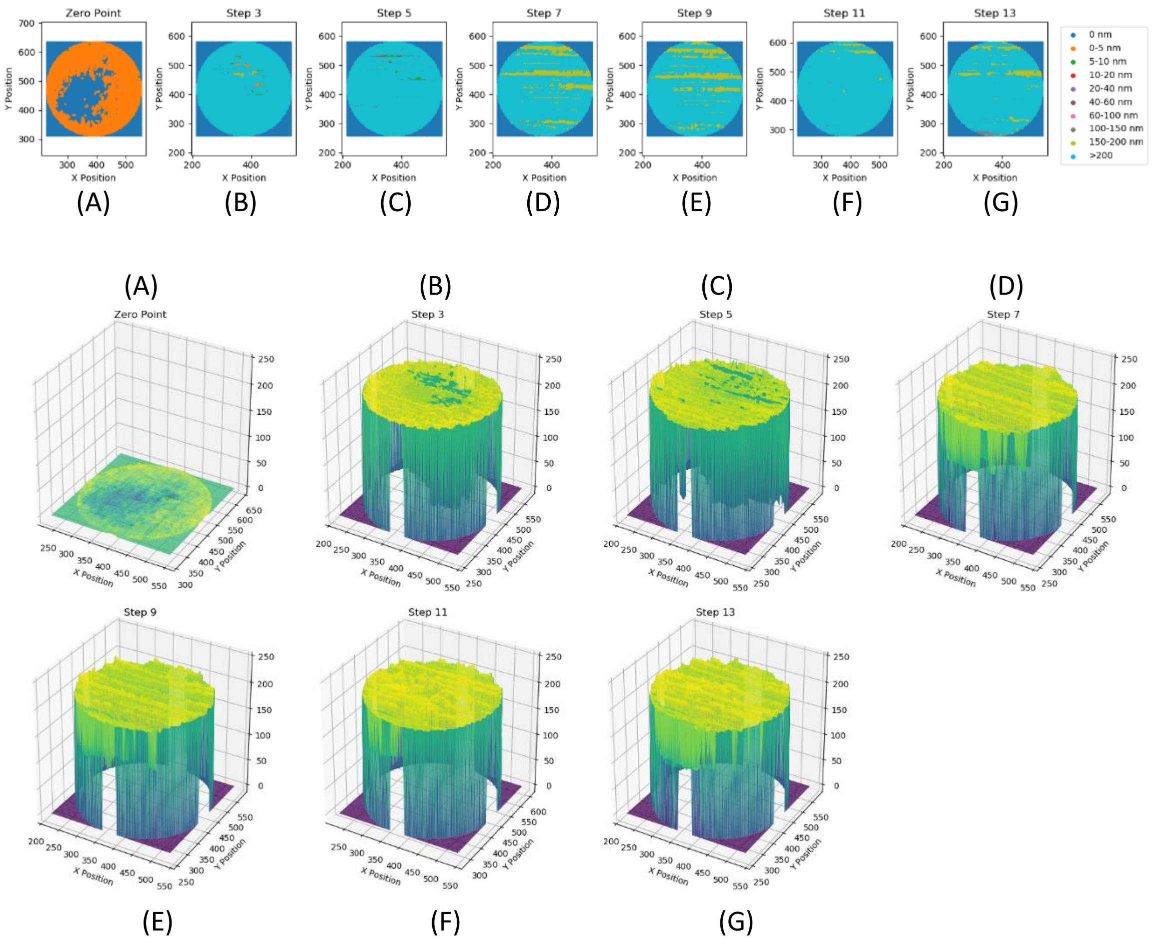

Figure S6. Plots of the film thickness (nm) for a 1 wt % block-like HB polymer and base oil formulation. Top: 2D scatter plots for each step, where the data has been binned, as indicated in the legend. Bottom: 3D surface plots for each step. The labels (A - G) correlate across the two plots and with the labels in Figure S4.

Additional Interpretation of Data in Figure S5

This may be due to the structure of the block-like polymer: due to the greater concentration of chain transfer agent (CTA) in the distilled oligomer mixture used to synthesise the block-like copolymer compared to the semi-block-like polymer, the block-like copolymer will possess a greater density of vinyl end groups. The crosslinking of these end groups is likely the origin of the observed film formation, hence if the block-like polymer possesses a greater density of end groups, the cross-link density within the film formed will also be greater, potentially leading to a more robust film being formed more quickly.

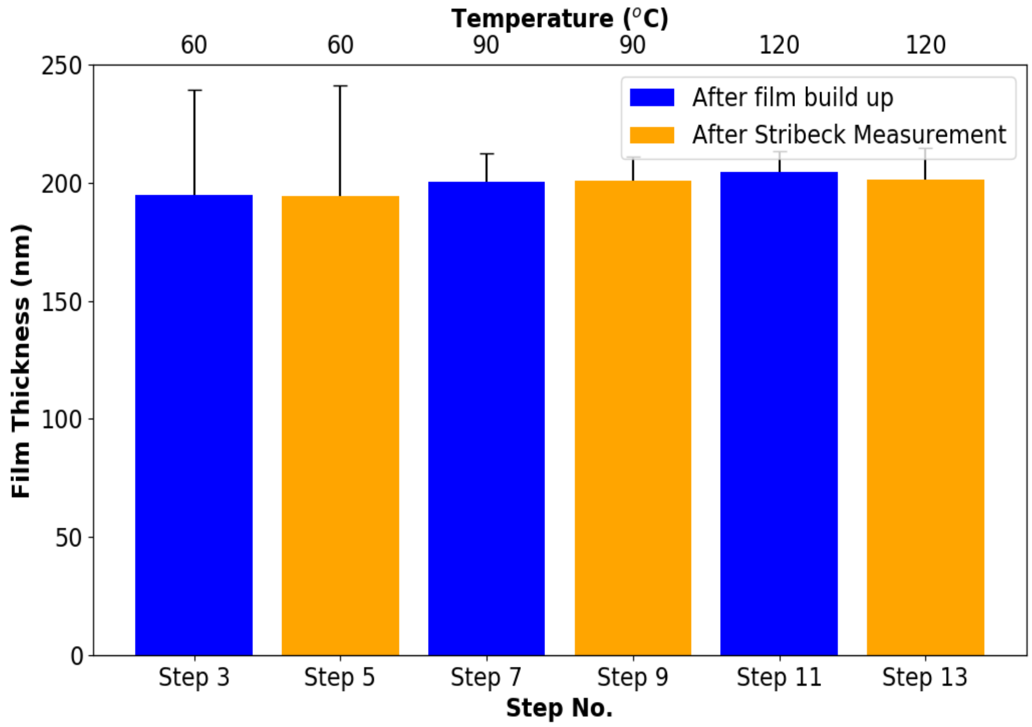

Figure S7. A visual representation of this data of Table 5, Entries B-G. It contains the plot of the film thickness in nm for these entries. The overall data in Figure S4 and Table 5 represent the Summary of the film thickness data obtained from interferometry for a 1 wt % block HB polymer/base oil formulation. The blue shading indicates that the measurement was made after film build up (i.e. following a period of constant temperature and rolling speed). The orange shading indicates that the measurement was made after a Stribeck Curve measurement was made. The plot (right) is a visual representation of this data (B-G).

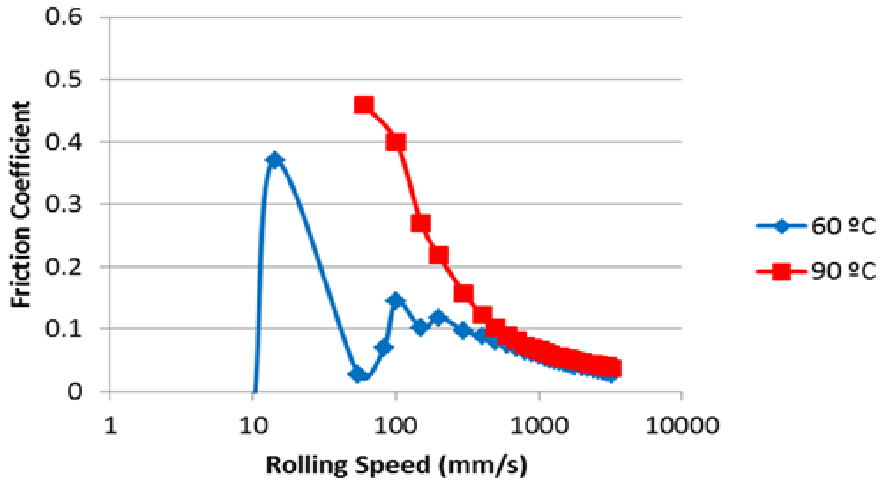

Figure S8. Stribeck curve of from an experiment using pure oil as the lubricant.

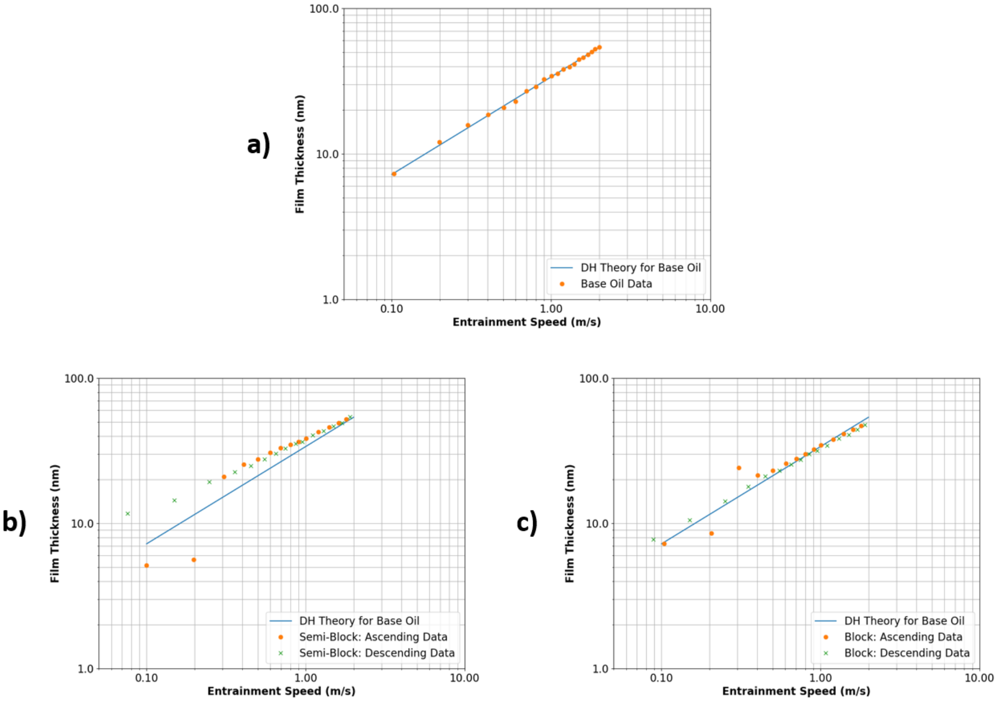

Figure S9. EHD data for a) PA04 Base oil, b) 0.5 wt% semi-block-like copolymer in solution and c) 0.5 wt% block-like copolymer in solution. The straight-line fit represents the theoretical values for an ideal (pure oil) solution. The circular data points represent data measured when the entraining speed was increasing, while the data point represented by ‘x’ show the data obtained when the entraining speed was decreasing.

Additional Data: Elastohydrodynamic Rig Testing

Previous testing focussed on boundary/very thin film conditions, which are the dominant regimes in an engine. However, rolling bearings are the second most used machine components. The small contact area and large pressures between rolling elements and raceways due to the non–conformity of their surfaces lead to very thin lubricant films, a regime of lubrication known as elastohydrodynamic (EHD).

Thus, the performance of the HB semi-block-like and block-like materials were tested in the EHD regime using 0.5 wt% polymer solutions. The film thickness was measured for each HB polymer using an EH rig with both ascending and descending entraining speed tests. The results are shown in FigureS8. The pure base oil was also measured for comparison.

For the PAO4, the data, shown in Figure S8a, demonstrated the expected variation in film thickness as a function of entraining speed. According to Dowson-Hamrock (DH) theory a plot of film thickness versus entraining speed should give a straight line of slope 0.67 when plotted with logarithmic axes,

which is known as ideal behaviour. This plot can be used to predict the film thickness at a given entraining speed, and is plotted as a straight line in Figurea. The experimental data for the base oil can be seen to closely match the theoretical data as expected.

For the semi-block-like copolymer, the data for which is shown in Figureb, two occurrences of non-ideal behaviour were observed. When running from low to high speed, at the two lowest speeds, the film thickness measured was smaller than expected for an ideal solution. This was probably due to the phenomenon of inlet rejection, where the polymer molecules were not fully solvated and were unable to enter the EHD contact. As the speed increased further, the polymer was able to enter the contact and the solution behaved more ideally. When running from high to low speeds, there was a notable increase in measured film thickness compared to that expected for an ideal solution. This indicated that the polymer from the solution had adsorbed onto the ball and/or disc, forming a protective film. It was also noted that the film thickness at an entrainment speed of 1 m/s was higher than that of the base oil alone, indicating a thickening effect of the polymer.

For the block-like copolymer, the data for which is shown in Figurec, the data was essentially the same as for the pure base oil. While a small amount of inlet rejection may be seen when entrainment speed is ascending, when descending there was no evidence of either non-ideal behaviour or increased oil film thickness at 1 m/s, indicating limited thickening effect of this polymer under these high stress high shear conditions. This is in contrast to the trend seen for the semi-block-like polymer, which may be attributed to the higher molecular weight of the semi-block-like polymer and its greater ability to act as a viscosity modifier.

At higher entrainment speeds both of the polymer-base oil solutions behaved as constant viscosity fluids, as evidenced by the fact that the gradients of their measurements were parallel to that of the base oil. This suggested shear-thinning of the polymers occurred at these increased entrainment speeds.
